# Absence of the celiac trunk and trifurcation of the common hepatic artery: a case report

**DOI:** 10.1590/1677-5449.004016

**Published:** 2016

**Authors:** Satheesha Nayak Badagabettu, Ashwini Aithal Padur, Naveen Kumar, Deepthinath Reghunathan

**Affiliations:** 1 Manipal University, Melaka Manipal Medical College, Department of Anatomy, Manipal, Karnataka, India.

**Keywords:** celiac trunk, common hepatic artery, gastric arteries, splenic artery, variation, tronco celíaco, artéria hepática comum, artérias gástricas, artéria esplênica, variação

## Abstract

Anatomical variations of the celiac trunk and its branches are particularly important from a surgical perspective due to their relationships with surrounding structures. We report here a particularly rare variant involving absence of the celiac trunk in association with trifurcation of the common hepatic artery. These variations were found in an adult male cadaver. We perform a review of the literature and discuss the clinical and embryological significance of these variations. Recognition of celiac trunk and hepatic artery variations is of utmost importance to surgeons and radiologists because multiple variations can lead to undue complications.

## Resumo

As variações anatômicas do tronco celíaco e seus ramos são particularmente importantes do ponto de vista cirúrgico devido a sua relação com as estruturas adjacentes. Nós descrevemos aqui uma variante particularmente rara envolvendo ausência do tronco celíaco associada a trifurcação da artéria hepática comum. Essas variações foram observadas no cadáver de um adulto do sexo masculino. Neste artigo revisamos a literatura e discutimos a significância clínica e embriológica dessas variações. O reconhecimento das variações do tronco celíaco e da artéria hepática é extremamente importante para cirurgiões e radiologistas uma vez que variações múltiplas podem levar a complicações inesperadas.

## INTRODUCTION

Knowledge of vascular variations in the abdominal region is important during various operative, diagnostic and endovascular procedures. The celiac trunk (CT) or celiac artery is the first ventral splanchnic branch of the abdominal aorta. It is the artery of the foregut and it originates from the ventral aspect of the abdominal aorta at the level of the junction between the T12 and L1 vertebrae. It is about 1.5-2 cm in length and usually terminates by dividing into three main branches: the left gastric, common hepatic and splenic arteries.^1^ Anatomical variations of the CT and its branches are particularly important from a surgical perspective because of their relationships with the surrounding structures. Reported variations of the CT include congenital absence, bifurcation and presence of collateral branches.^2^^-^ 4 However, absence of the CT is a very rare variation. Its incidence is reported to be as low as 0.2%.^5^ Bordei et al. reported such a variation in which all three branches of the celiac trunk arose from the aorta.^6^ Yuksel et al.^7^ reported a case of an inferior phrenic trunk arising from the celiac trunk. Other reported variations include: a common trunk of the left gastric and left inferior phrenic arteries, arising from the CT,^8^ hepatogastric and hepatosplenic trunks arising from the CT,^9^ and a middle colic artery with origin at the CT.^10^ Cicekcibasi et al. reported the occurrence of a celiacomesenteric trunk, which gave rise to the left gastric, common hepatic, splenic, left gastro-epiploic, and right and left inferior phrenic arteries.^11^ Nayak reported the presence of a celiaco-mesenterico-phrenic trunk, which was long and trifurcated to give rise to a celiac trunk, the superior mesenteric artery, and an inferior phrenic trunk.^12^ Clinicians should be aware of such variations before performing angiographic examinations and laparoscopic surgeries for celiac axis compression syndrome. The common hepatic artery (CHA) is a branch of the celiac artery to the point where the gastroduodenal artery arises, beyond which it becomes the proper hepatic artery. Reports have shown that the CHA can have a variant origin and can have a different anatomic course, but variation in the branching pattern of the common hepatic artery is rare. Song et al. conducted an extensive study of the common hepatic artery and found variations in only 3.71% of cases.^13^ Variations in the anatomy of hepatic arteries are being increasingly documented in anatomical, radiographic, and surgical literature. Although the incidence of these arterial variations has been recorded, rare and unique variations continue to be discovered both in cadaveric dissections and in clinical settings. In this paper, we report a particularly rare variation involving absence of the celiac trunk associated with trifurcation of the common hepatic artery and discuss its embryological basis and clinical significance.

## CASE REPORT

**Figure 1:**
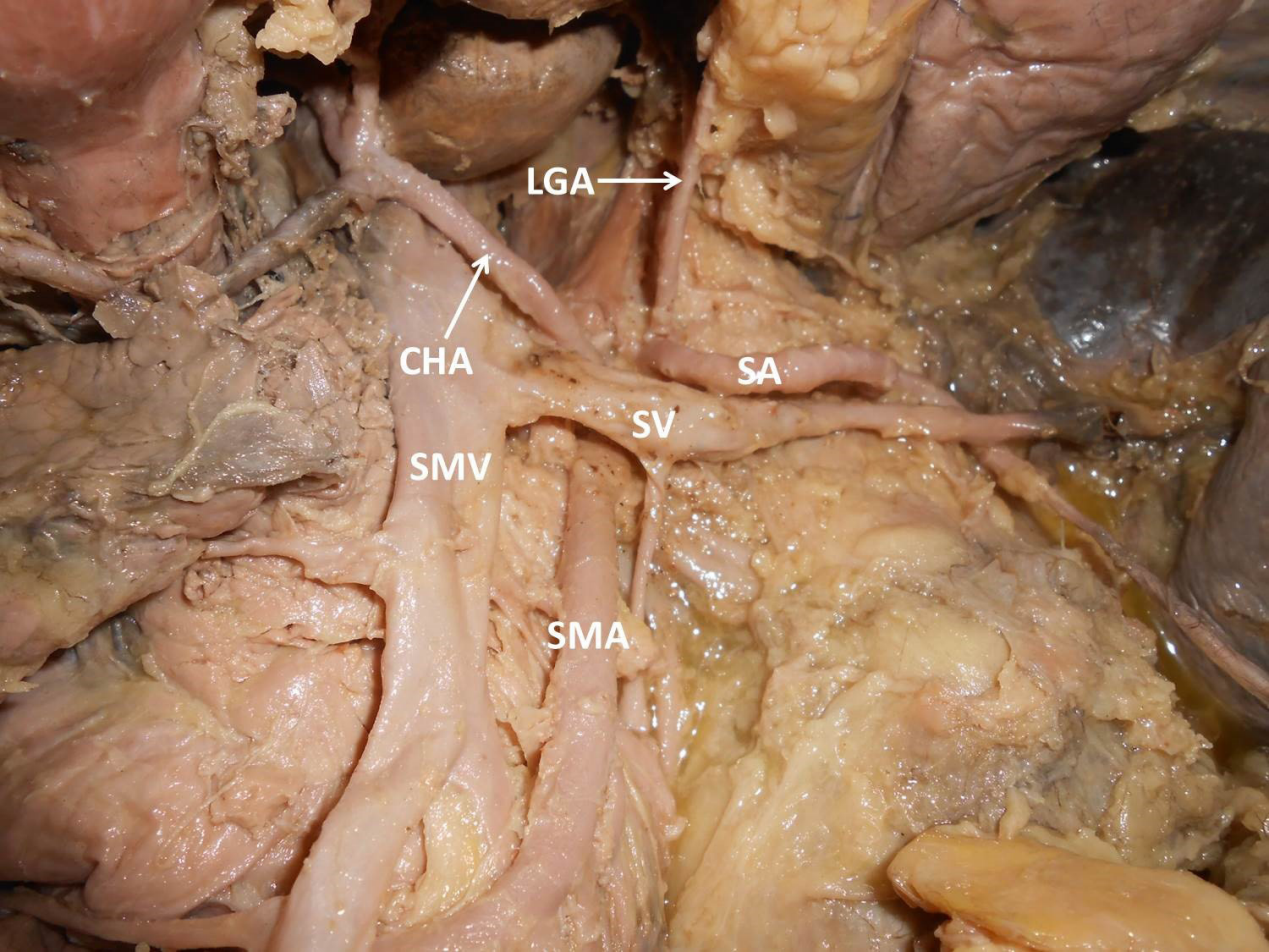
Photograph of dissection of the upper abdominal vessels. CHA: common hepatic artery; LGA: left gastric artery; SA: splenic artery; SMA: superior mesenteric artery; SMV: superior mesenteric vein; SV: splenic vein.

**Figure 2:**
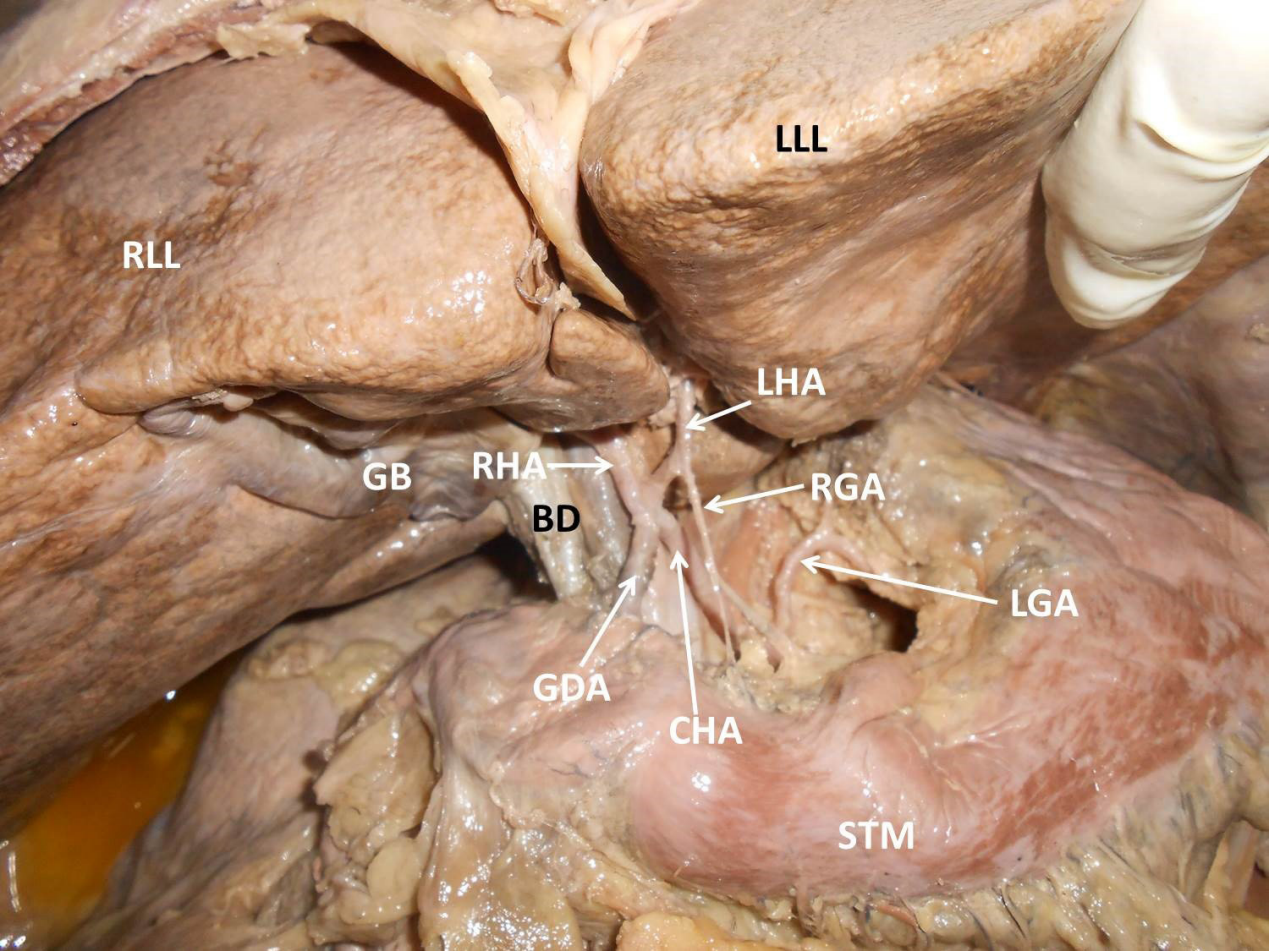
Photograph showing the variant branching pattern of upper abdominal vessels. BD: bile duct; CHA: common hepatic artery; GB: gall bladder; GDA: gastroduodenal artery; LGA: left gastric artery; LHA: left hepatic artery; LLL: left lobe of liver; RGA: right gastric artery; RHA: right hepatic artery; RLL: right lobe of liver; STM: stomach.

**Figure 3:**
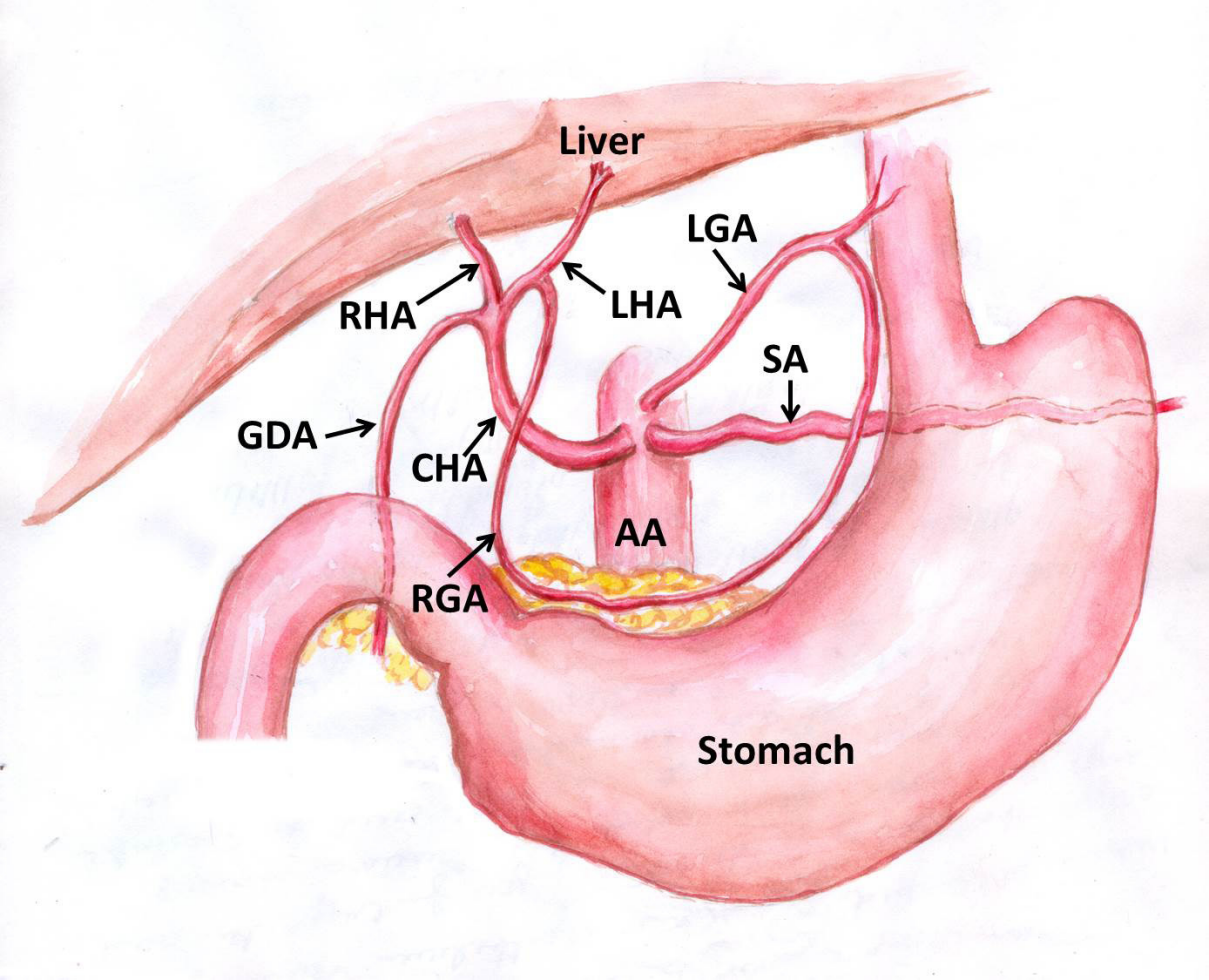
Schematic diagram showing absence of the celiac trunk and trifurcation of the common hepatic artery. AA: abdominal aorta; CHA: common hepatic artery; GDA: gastroduodenal artery; LGA: left gastric artery; LHA: left hepatic artery; RGA: right gastric artery; RHA: right hepatic artery; SA: splenic artery.

During dissection classes for medical undergraduates, we observed the following variations of abdominal blood vessels in an adult male cadaver aged approximately 60-65 years, with a height of approximately 1.65 m, and body weight of 60 kg. The celiac trunk was absent. The left gastric artery, splenic artery and common hepatic artery all arose directly from the abdominal aorta (Figure 1). The superior mesenteric artery had a high origin, very close to the three arteries specified above. The splenic artery and left gastric artery had normal course and distribution. However, the common hepatic artery had a variant branching pattern. The common hepatic artery coursed upwards and to the right and trifurcated into the right hepatic, left hepatic and gastroduodenal arteries. This resulted in absence of the hepatic artery proper (Figure 2). The common hepatic artery trifurcated 2 cm above the first part of the duodenum. The right gastric artery arose from the left hepatic artery (Figure 3). The left hepatic artery entered the liver through the fissure for the ligamentum venosum. The right hepatic artery had a normal course.

## DISCUSSION

Anatomic variations of the celiac trunk and its branches are the result of anomalous embryogenesis of primitive ventral blood vessels originating from the abdominal aorta. Each dorsal aorta gives off ventral splanchnic arteries which supply the gut and its derivatives. Initially, these ventral branches are paired, but with the fusion of the dorsal aorta, they also fuse to form a series of unpaired segmental arteries which run in the dorsal mesentery of the gut. They gradually fuse to form the arteries of the foregut, midgut, and hindgut.^14^ There are four ventral segmental arteries arising from the dorsal aorta in early human embryos and these arteries are connected to the ventral longitudinal anastomosis. During the development process, the primitive arteries converge into three arteries which correspond to the celiac trunk, superior mesenteric artery, and inferior mesenteric artery. In the case of absence of celiac trunk, the longitudinal anastomoses regress completely. However, the roots of the ventral segmental arteries do not regress. The 10th primitive root of the ventral segmental artery becomes the left gastric artery; the 11th becomes the splenic artery; the 12th becomes the common hepatic artery; and the 13th and 19th become the superior mesenteric artery and inferior mesenteric artery respectively, with separate origins from the abdominal aorta.^15^ This may be the embryological basis of these types of variations.

Absence of the celiac trunk is a rare anomaly with incidence rates ranging from 0.1^16^ to 2.6%.^17^ According to Iacob et al.,^18^ only 31 cases of absent celiac trunk have been reported worldwide and about 1/3 of these cases were detected by imaging studies, while the remainder of these variations were noticed during anatomical dissections. Morita has proposed a classification of types of celiac trunk as follows: (i) normal celiac trunk; (ii) hepatosplenic trunk; (iii) gastrosplenic trunk; (iv) hepatogastric trunk; and (v) absent celiac trunk.^19^ The variation reported herein is of the fifth type in Morita’s classification and is considered to be very rare. Lee et al. reported a case of an incomplete celiac trunk in which the left gastric artery, the splenic artery, and the hepatomesenteric trunk all arose independently from the abdominal aorta.^20^

Knowledge of anatomical variations of the celiac trunk is important for surgeons during liver transplantation, laparoscopic surgery, radiological abdominal interventions, and penetrating injuries to the abdomen. Also, acquaintance of unique variations of absence of the celiac trunk may be useful in planning and performing radiological interventions such as celiacography^21^ and chemoembolization of hepatic tumors^22^ and so such arterial variations are very important and must not be ignored. Celiac artery variations are said to increase both the difficulty and the risk of radical gastrectomy.^23^

Another variation that we report here is trifurcation of the common hepatic artery. Although many reports have been published on variant branches of the hepatic artery, its trifurcation is uncommon. Nayak et al.^24^ reported such an occurrence of trifurcation of the common hepatic artery and in their opinion it is very rare. Surgeons who perform liver transplants, laparoscopic surgery, pancreatic mobilizations, and gastrojejuno-stomies, and also radiologists attending abdominal interventions should be mindful of variations of the hepatic arteries to avoid inadvertent or iatrogenic hepatic vascular injury. When there are multiple variations, as in the case reported herein, the chance of vascular damage is very high. At present, many interventional and new surgical techniques have been developed to treat both primary and metastatic tumors and due to the increasing availability of living-related liver transplant donors, accurate depiction of hepatic and celiac arterial anatomy is important because it enables surgeons to accurately plan for a resection, expedites the operative procedure, and helps to avoid inaccuracies during ligation of vessels, which might lead to severe postoperative morbidities, bilomas, hematomas, sepsis or even mortality.^25^

## CONCLUSION

Recent advances in imaging studies have made accurate evaluation of the vascular anatomy of the upper gastrointestinal tract easier. Recognition of celiac trunk and hepatic artery variants is of utmost importance because it aids in planning of several surgical and interventional procedures, thereby helping to avoid undue complications.
